# Diagnostic Discordance During MDR-TB Treatment: Retrospective Identification of *Mycobacterium avium Complex*-Associated Nontuberculous Mycobacterium by Whole-Genome Sequencing

**DOI:** 10.3390/microorganisms14071596

**Published:** 2026-07-22

**Authors:** Ivy Rukasha, Kabelo Gabriel Kaapu, Nakamozi Francine Nemaguvhuni, Felicia Wells-Hunter, Abhinav Sharma, Jody Emile Phelan, Mutlisi Jacqueline Kolobe, Molebogeng Ruth Lekalakala-Mokaba, Robin Mark Warren, Emilyn Costa Conceição

**Affiliations:** 1 Division of Medical Microbiology, Department of Pathology, School of Medicine, University of Limpopo, Polokwane 0727, South Africa; 2 South African Medical Research Council Centre for Tuberculosis Research, Division of Molecular Biology and Human Genetics, Faculty of Medicine and Health Sciences, Stellenbosch University, Cape Town 7505, South Africaemilyncosta@sun.ac.za (E.C.C.); 3 Multidrug Resistant Tuberculosis Unit, Tshilidzini Hospital, Limpopo Department of Health, Limpopo 0950, South Africa; 4 Department of Infection Biology, Faculty of Infectious and Tropical Diseases, London School of Hygiene and Tropical Medicine, Keppel Street, London WC1E 7HT, UK; 5 Department of Microbiology, National Health Laboratory Service, Polokwane 0699, South Africa; 6 Centre for Epidemic Response and Innovation (CERI), School for Data Science and Computational Thinking, Stellenbosch University, Cape Town 7505, South Africa

**Keywords:** nontuberculous mycobacteria, multidrug-resistant tuberculosis, Limpopo

## Abstract

Nontuberculous mycobacteria (NTM) pose significant diagnostic challenges in high tuberculosis (TB)-burden settings, particularly when routine molecular assays suggest multidrug-resistant TB (MDR-TB). Current diagnostic algorithms in high TB-burden settings are primarily designed to detect members of the *Mycobacterium tuberculosis* *complex* (MTBC) and may inadequately distinguish NTM when discordant laboratory findings are encountered. The value of this case lies not in demonstrating that whole-genome sequencing (WGS) should guide real-time treatment, but in illustrating how diagnostic uncertainty can arise when routine MTBC-focused tests fail to identify NTM and how genomic surveillance can contribute to species-level characterization in such settings. We report a complex case which was initially diagnosed as rifampicin- and isoniazid-resistant MTBC and subsequently managed according to programmatic MDR-TB guidelines. Persistent acid-fast bacilli positivity in the presence of negative MTBC antigen testing during follow-up raised suspicion of NTM involvement. Whole-genome sequencing (WGS), performed retrospectively on a follow-up isolate, identified an NTM belonging to the *Mycobacterium avium complex* (MAC) which was phylogenetically closest to genomes provisionally designated *Mycobacterium europaeum_A* and distinct from *Mycobacterium europaeum* sensu stricto, a member of the *Mycobacterium simiae complex*. The identification of a MAC-associated NTM provided an explanation for the observed diagnostic discordance but could not determine whether the organism represented colonization, sequential infection, or concurrent infection. Although WGS did not inform clinical management, it provided high-resolution species identification and highlighted important limitations of MTBC-focused diagnostic workflows when discordant microbiological findings are encountered. This case underscores the need for the targeted investigation of NTM in patients with acid-fast bacilli-positive cultures and negative MTBC antigen testing and supports the use of genomic surveillance to improve species-level identification and understanding of NTM epidemiology in high TB-burden settings such as Limpopo Province, South Africa.

## 1. Introduction

Nontuberculous mycobacteria (NTM) are increasingly recognized as important causes of pulmonary disease, particularly in high tuberculosis (TB)-burden settings where they frequently mimic *Mycobacterium tuberculosis* complex (MTBC) clinically and microbiologically [1,2]. In regions where TB remains endemic, smear positivity, or compatible radiological findings often trigger immediate molecular testing and empiric treatment, creating a diagnostic environment heavily oriented toward MTBC detection [3,4].

Routine diagnostic workflows typically include smear microscopy, GeneXpert MTB/RIF Ultra (Cepheid, Sunnyvale, CA, USA), liquid culture (e.g., BD BACTEC™ MGIT™ system), and line probe assays (LPAs) such as GenoType MTBDRplus, GenoType MTBDRsl, and GenoType CM (Hain Lifescience GmbH, Nehren, Germany) [5]. While highly valuable for the rapid detection of MTBC and associated resistance mutations, these assays are not designed for comprehensive NTM species identification. Cross-reactivity, the amplification of conserved genomic regions, and the intrinsic polymorphisms in NTMs may result in misleading MTBC detection or false resistance patterns [6].

Diagnostic complexity increases further in the context of potential mixed infection with MTBC and NTM. In such cases, discordant laboratory findings may occur, including positive molecular resistance signals alongside negative antigen tests or atypical culture behaviour [7]. Distinguishing true multidrug-resistant TB from NTM disease or merely NTM colonization requires careful clinical correlation, repeated microbiological confirmation, and species-level identification, resources not consistently available in high-burden TB routine settings [8,9].

Accurate NTM identification is clinically relevant because antimicrobial susceptibility patterns vary substantially across mycobacterial complexes. However, most molecular assays used in TB programmes do not provide definitive taxonomic resolution [10,11]. Whole-genome sequencing (WGS) offers comprehensive species identification and can detect genomic divergence, mixed populations, and heteroresistance, yet it is rarely incorporated early in diagnostic algorithms in high-burden regions [12].

Here, we present a case from Limpopo Province, South Africa, which was initially diagnosed and managed as multidrug-resistant tuberculosis based on routine molecular testing. The retrospective whole-genome sequencing of a follow-up isolate identified a *Mycobacterium avium complex* (MAC)-associated NTM and revealed important diagnostic limitations within conventional MTBC-focused workflows. Rather than demonstrating the role of WGS in directing patient management, this case highlights the need for species-level identification in patients with discordant laboratory findings and illustrates the potential value of genomic surveillance for improving understanding of NTM epidemiology in high TB-burden settings.

## 2. Case Presentation

A 49-year-old, Black African, HIV-negative, immunocompetent male from Vhembe District, Limpopo Province (bordering Botswana, Zimbabwe, and Mozambique), presented with a three-month history of productive cough, low-grade fever, night sweats, weight loss, and fatigue. He had no prior history of TB, chronic lung disease, or immunosuppression. He was a smoker, denied alcohol or illicit drug use, and worked in agriculture with regular soil and water exposure (Table 1).

**Figure 1:**
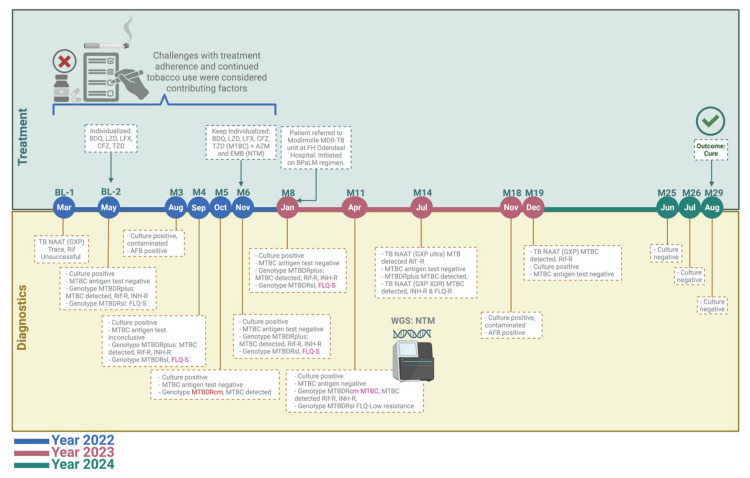
Laboratory and clinical diagnostic investigation timeline.

On examination, he was mildly cachectic but afebrile and in no acute distress. Chest auscultation revealed bilateral coarse crackles, predominantly in the upper lobes; oxygen saturation was normal. Baseline chest X-ray demonstrated the complete opacification of the left lung, without documented cavitary disease. Given the high local TB burden and classic constitutional symptoms, pulmonary TB was strongly suspected, and sputum was submitted for a diagnostic investigation that followed the diagnostic workflow in Figure 1.

### 2.1. Drug Susceptibility Testing, Disease Progression and Clinical Course

Initial laboratory testing using Xpert MTB/RIF Ultra detected MTBC with rifampicin (RIF) resistance. Sputum smear microscopy revealed scanty acid-fast bacilli (AFB), and MGIT culture confirmed AFB growth; however, the MTBC antigen test was negative at that stage. This discordance, an AFB-positive culture with a negative MTBC antigen test, represents an important diagnostic red flag suggestive of NTM and should prompt early species-level identification. Further molecular characterization with GenoType CM and GenotyType MTBDRplus diagnosed MTBC with resistance to both rifampicin (RIF) and isoniazid (INH), while MTBDRsl demonstrated susceptibility to fluoroquinolones. Based on these findings, the patient was initiated on multidrug-resistant tuberculosis (MDR-TB) treatment.

In 2022, following molecular confirmation of RIF and INH resistance, an individualized longer MDR-TB regimen was commenced, consisting of bedaquiline, linezolid, levofloxacin, clofazimine, and terizidone. At baseline, sputum smear microscopy remained AFB positive and TB culture positive. During follow-up, microscopy demonstrated a heavy bacillary load (3+), and repeat molecular testing showed persistent MTBC detection. Xpert XDR subsequently indicated low-level fluoroquinolone resistance, suggesting progression to pre-XDR-TB. Treatment was modified accordingly; however, later testing confirmed full fluoroquinolone resistance, consistent with further disease progression.

During months 5–6 of treatment, in the context of persistent non-MTBC growth and clinical suspicion of NTM involvement, azithromycin and ethambutol were added to broaden antimicrobial coverage. By month 7, sputum AFB smear and TB culture converted to negative. However, at month 8, sputum reverted to AFB positive and culture positive. Treatment interruption and tobacco use were documented as contributing factors during this period. Following the recurrence of culture positivity, the patient was referred to the Modimolle MDR-TB unit in 2023, where he was transitioned to the BPaLL (bedaquiline, pretomanid, linezolid, and levofloxacin) regimen in accordance with national programmatic guidelines. The patient subsequently completed treatment successfully, achieving sustained sputum culture conversion to negative, with follow-up extending into 2024.

### 2.2. Advanced Investigation Using Whole-Genome Sequencing

The cryopreserved isolate (Ref: UL-13) from the isolate collected on April/2023 was sub-cultured using the BD BACTEC MGIT™ system (Becton, Dickinson and Company, Franklin Lakes, NJ, USA) with a growth supplement and PANTA. After culture positivity, the sample was incubated for two additional weeks at 37 °C to increase biomass. Quality control included Ziehl–Neelsen staining and blood agar to confirm AFB presence and purity. The total culture content (about 7 mL) was heat-inactivated at 80 °C for 1 h and shipped to Stellenbosch University at the Biosafety Level (BSL)-3 laboratory.

The total volume of heat-inactivated culture was transferred to a 15 mL Falcon tube and transported to a BSL-2 laboratory. DNA extraction followed the InstaGene Matrix (Bio-Rad Laboratories, Hercules, CA, USA) with FastPrep-24™ (MP Biomedicals, Irvine, CA, USA) protocol by Conceição et al., 2024 [13]. DNA and library quantification were performed using Qubit High-Sensitivity (HS) assays, and fragment size distribution was assessed using the Agilent 4150 TapeStation system (Agilent Technologies, Santa Clara, CA, USA). Extracted DNA was quantified using the Qubit HS double-stranded DNA (dsDNA) assay kit (Thermo Fisher Scientific, Waltham, MA, USA), yielding a concentration of 1.11 µg/µL. Libraries were prepared using the Illumina DNA Prep kit, resulting in a final library concentration of 4.76 µg/µL with an average fragment size of 458 bp. Sequencing was performed on the Illumina MiniSeq platform and high output reagent kit, targeting approximately 50× genome coverage.

Initial bioinformatics analysis used the MAGMA pipeline (https://github.com/TORCH-Consortium/MAGMA) [14]. Read quality was assessed using FastQC (v0.11.9), and low-quality reads and adapter sequences were trimmed using Trimmomatic (v0.39). Filtered reads were aligned to the *Mycobacterium tuberculosis* H37Rv reference genome using standard alignment workflows, and downstream processing was performed with SAMtools (v1.17) and Picard (v3.0.0). Further investigation included NTM-Profiler v0.7.0 (https://github.com/jodyphelan/NTM-Profiler) which estimated genome similarity and relative abundance directly from sequencing reads using sylph. This was performed using the curated ntmdb reference database (commit 8feb8271) which contains 2910 sequences across 256 unique taxa as assigned by the Genome Taxonomy Database (GTDB) (v220). WGS was performed retrospectively for research purposes and did not inform real-time clinical decision-making.

## 3. Results and Discussion

This case illustrates the substantial diagnostic challenges posed by NTM in high TB-burden settings, particularly when routine molecular assays suggest MDR-TB. The patient was managed according to programmatic MDR-TB guidelines following clinical and microbiological tests. Persistent MTBC antigen negativity in the presence of AFB positive raised suspicion of an NTM organism. This case highlights that discordant diagnostic findings, particularly the combination of an AFB-positive culture with negative MTBC antigen testing, should be recognized as a critical diagnostic red flag for NTM. In high TB-burden settings, where diagnostic workflows are heavily oriented toward the detection of MTBC, such discordance may be overlooked or attributed to technical variability, potentially delaying appropriate investigation. Early recognition of this pattern should prompt timely species-level identification using targeted assays or genomic approaches, thereby improving diagnostic accuracy and supporting more appropriate clinical decision-making. The sequence was provisionally annotated as *Mycobacterium europaeum*_A, as defined by GTDB, using an NTM-profiler and mashtree. The *Mycobacterium europaeum*_A clade sits within the *Mycobacterium avium complex* (MAC) clade rather than alongside *M. europaeum* sensu stricto of the *Mycobacterium simiae* complex.

Compared to previously reported NTM cases, the novelty of this report lies in the presence of diagnostic discordance, specifically an AFB-positive culture with negative MTBC antigen testing, leading to its misclassification as MDR-TB based on molecular assays. Additionally, the retrospective identification of a MAC-associated NTM during ongoing MDR-TB treatment highlights a critical gap in routine diagnostic workflows in high TB-burden settings. Importantly, this case demonstrates that favourable clinical outcomes may occur despite unresolved diagnostic uncertainty, underscoring the need for improved species-level identification strategies.

The timing of WGS relative to treatment milestones is equally important for narrative clarity. The sequenced sample corresponded to a period of persistent NTM during follow-up rather than at initial presentation. Therefore, genomic findings reflect the microbiological landscape at that specific timepoint and do not allow for the determination of whether the patient had mixed infection at baseline, sequential infection, or NTM colonization emerging during treatment. This temporal context is essential when interpreting whether the patient experienced sequential infection, true mixed infection, or the replacement of MTBC by NTM during therapy. Understanding the treatment regimen at each diagnostic stage further contextualizes how molecular results influenced clinical decisions and highlights how earlier genomic clarification might have altered management [15].

WGS identified an NTM that was genomically distinct from classical reference strains and clustered near previously misannotated genomes, such as *M. europaeum*_A, (GCF_001673415.1, ANI result of 99.18 and abundance of 99.525) which clustered within the MAC rather than with *M. europaeum* sensu stricto (Supplementary File S1), a rare slow-growing scotochromogenic species belonging to the *M. simiae* complex that was proposed as a novel species following the polyphasic analyses of clinical strains recovered from European patients [16,17]. Supplementary File S1 contains the mash distance tree and genomic similarity analysis supporting species classification. If WGS was used to guide patient care, this reclassification would have direct clinical implications, as MAC-associated organisms require fundamentally different therapeutic approaches compared to *M. simiae* complex or MDR-TB [18]. The isolate clustered with genomes was provisionally annotated in public databases, suggesting ongoing taxonomic complexity within this group; however, further genomic and phenotypic characterization would be required to determine its precise classification.

In this case report, besides the MDR-TB treatment, azithromycin and ethambutol were added to the patient treatment, which may have covered potential mixed populations as according to Clinical and Laboratory Standards Institute (CLSI) guidance for MAC pulmonary disease. Standard therapy for macrolide-susceptible strains typically includes a macrolide (azithromycin or clarithromycin) combined with ethambutol, with the addition of rifamycin’s or aminoglycosides depending on disease severity and resistance patterns [19,20]. In this case, rifampicin was resistant based on GeneXpert Ultra and LPA Genotype MTBDRplus; however, rifampicin resistance in MTBC-directed assays may not directly translate to rifamycin resistance in MAC, confirmed resistance would warrant the revision of therapy and the consideration of alternative agents, potentially including amikacin, particularly in severe disease [6,21]. Importantly, WGS in this study was performed for research purposes and did not inform real-time clinical decision-making, yet its findings underscore the potential impact of early genomic resolution in similar scenarios.

While mixed infection with MTBC and NTM is a recognized phenomenon, this case does not provide sufficient evidence to confirm its presence, as sequencing was not performed on baseline isolates. Molecular assays such as LPA GenoType CM are designed to detect clinically relevant NTMs but have a limited panel. In addition, liquid culture systems may preferentially recover NTMs, while antigen-based tests remain negative, creating apparently contradictory laboratory results. In such contexts, differentiating true MDR-TB from NTM disease, transient colonization, or mixed infection becomes highly complex and may lead to prolonged exposure to toxic second-line regimens [22,23].

The clinical relevance of NTM isolation further complicates management. NTMs are ubiquitous environmental organisms, and isolation from respiratory specimens does not necessarily equate to active disease [24]. Establishing pathogenic significance requires the integration of clinical, radiological, and microbiological criteria, including repeated culture positivity [25]. In this case, prolonged anti-TB treatment and evolving diagnostic findings make it difficult to definitively distinguish sequential infection, potential mixed infection, or colonization. Nevertheless, the genomic findings demonstrate that reliance solely on MTBC-targeted molecular tools may be insufficient in cases with discordant or atypical patterns [24,25].

Although WGS in this study was performed retrospectively for research purposes and did not guide real-time patient care, it demonstrates several advantages that support earlier integration into diagnostic workflows. WGS provides high-resolution species identification beyond the capacity of LPAs, detects genomic divergence from reference strains, and can reveal mixed infections or heteroresistance. In complex cases with discordant molecular findings, antigen-negative cultures, unexpected treatment responses, or early genomic investigation could prevent unnecessary escalation to toxic second-line TB regimens and enable pathogen-directed therapy aligned with international guidelines [26].

This case therefore illustrates not only the risk of misclassifying NTM as drug-resistant TB, but also the therapeutic consequences of delayed species-level identification. It supports a targeted implementation strategy for WGS in high-burden settings, particularly for LPA-discordant samples, persistent culture-positive cases, and suspected mixed infections [18]. Building on this pilot investigation, we aim to expand genomic analysis of NTM-indicative samples in Limpopo Province to better define local epidemiology, refine diagnostic algorithms, and integrate precision genomic tools into regional mycobacterial care.

When applying ATS/IDSA criteria for NTM pulmonary disease, this case does not fulfil the requirements for confirmed NTM disease. Only a single NTM isolate was identified, without repeated microbiological confirmation, and the patient demonstrated clinical improvement on a regimen lacking activity against MAC organisms. These findings suggest that the NTM detected may represent colonization rather than active disease.

Notably, the patient achieved a clinical cure following treatment with the BPaLL regimen, which does not provide effective coverage for MAC organisms. This further supports the interpretation that the detected NTM was unlikely to represent the primary pathogenic organism. Although the patient outcome was favourable, this case illustrates that correct treatment does not necessarily equate to correct diagnosis, highlighting an important gap in diagnostic accuracy.

Although WGS did not influence treatment decisions in this case, the report remains clinically and scientifically relevant for several reasons. First, it demonstrates that the combination of an AFB-positive culture and negative MTBC antigen testing should be recognized as an important diagnostic red flag for NTM, prompting further species-level investigation. Second, it provides evidence of NTM circulation in Limpopo Province, a region where the epidemiology, diversity, and clinical significance of NTM remain poorly characterized. Finally, it highlights the potential value of genomic surveillance and species-level identification in refining future diagnostic algorithms. While WGS was applied retrospectively in this case, its use in similar diagnostically challenging scenarios may help reduce misclassification, improve understanding of local mycobacterial epidemiology, and support more accurate management of patients presenting with discordant laboratory findings.

**Figure 2:**
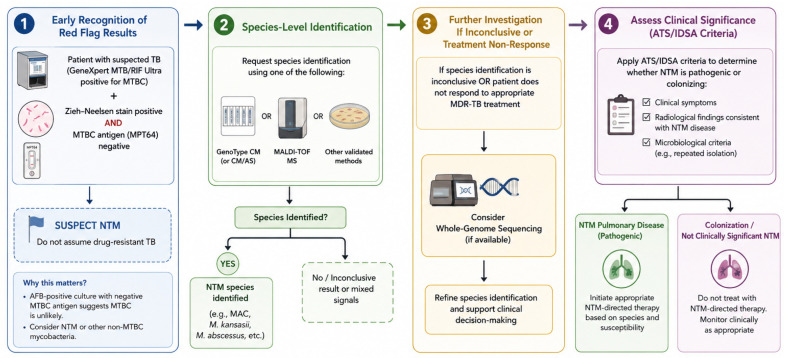
Proposed diagnostic algorithm for investigating discordant mycobacterial results in high TB-burden settings. Algorithm is based on lessons learned from present case and recommends early investigation for NTM when AFB-positive cultures are accompanied by negative MTBC antigen testing. Species-level identification should be performed using available methods such as GenoType CM/AS or MALDI-TOF MS, while WGS may be considered in unresolved or diagnostically challenging cases. Clinical significance should be determined using ATS/ERS/ESCMID/IDSA diagnostic criteria for NTM pulmonary disease.

Based on the lessons learned from this case, we propose a pragmatic diagnostic algorithm for investigating discordant mycobacterial results in high TB-burden settings (Figure 2). The algorithm emphasizes the early recognition of discordant findings, particularly an AFB-positive culture in combination with negative MTBC antigen testing, followed by stepwise species-level identification and clinical assessment. Such an approach may facilitate earlier recognition of NTM, reduce diagnostic uncertainty, and minimize the risk of its misclassification as drug-resistant tuberculosis.

**Key limitation for clinical relevance.** Whole-genome sequencing was performed retrospectively and did not influence treatment decisions in this case. The patient was managed according to standard MDR-TB treatment guidelines and achieved a favourable clinical outcome before genomic findings became available. Furthermore, the patient demonstrated clinical improvement despite the absence of targeted therapy against MAC, suggesting that the detected NTM may not have been the primary driver of disease. Consequently, this case should not be interpreted as evidence supporting routine WGS for all patients with suspected drug-resistant tuberculosis. Rather, it supports the selective use of species-level identification and genomic investigation in patients with diagnostic discordance, persistent culture positivity, unexpected treatment response, or suspected NTM involvement.

**General study limitations.** Several limitations should be considered when interpreting this case. First, WGS was performed only on a follow-up isolate and not on the initial diagnostic specimen. Consequently, it was not possible to determine whether the detected NTM represented a concurrent infection present at baseline, a sequential infection acquired during treatment, or transient colonization. Second, the absence of baseline genomic data precluded investigation of potential interactions between MTBC and NTM during the course of the disease. Third, microbiological confirmation was limited to a single sequenced NTM isolate, preventing the assessment of the persistence or repeated recovery of the same organism. Finally, additional clinical and inflammatory markers, including T-SPOT, erythrocyte sedimentation rate (ESR), and high-sensitivity C-reactive protein (hsCRP), were not available from the retrospective clinical record, limiting further clinical correlation and assessment of disease activity.

## 4. Conclusions

This case highlights important limitations of current mycobacterial diagnostic algorithms in high TB-burden settings. Although retrospective WGS identified a MAC-associated NTM, it did not influence clinical management and cannot determine whether the organism represented colonization, sequential infection, or a concurrent infection. Rather than demonstrating a role for retrospective WGS in successfully treated patients, this case underscores the need for improved approaches to investigating discordant mycobacterial results. In particular, the combination of an AFB-positive culture and negative MTBC antigen testing should prompt targeted investigation for NTM. Furthermore, the detection of a MAC-associated NTM highlights the need for prospective genomic surveillance to better understand the epidemiology, diversity, and clinical significance of NTM in Limpopo Province. Future surveillance studies incorporating species-level identification and the genomic characterization of discordant mycobacterial cultures may help refine diagnostic algorithms and reduce the misclassification of mycobacterial disease in South Africa.

## Figures and Tables

**Table 1:** Case presentation summary.

Parameter	Observation
Age/Sex	49-year-old male.
Ethnicity	Black African.
HIV Status	Negative.
Immunological Status	Immunocompetent.
Occupation	Agricultural worker (soil/water exposure).
Symptoms	Cough (3 months), night sweats, fatigue, weight loss.
Chest X-Ray	Complete opacification of the left lung, no cavitary disease.
Comorbidities	Not reported.
Smoking	Reported.
Alcohol or Illicit drug use	Not reported.
Prior TB history	None.
Initial Diagnosis	Multidrug-resistant tuberculosis (MDR-TB) (based on molecular assays).
Final Diagnosis	MDR-TB with subsequent detection of *Mycobacterium avium complex* (MAC)-associated (clinical significance uncertain: colonization vs. transient NTM recovery, while sequential or mixed infection cannot be excluded).
Outcome	Cure

## Data Availability

The raw fastq files were submitted to the European Nucleotide Archive (ENA) Bioproject ID: PRJEB104875 and Biosample Id ERS28338218.

## Supplementary Materials

The following supporting information can be downloaded at: https://www.mdpi.com/article/10.3390/microorganisms14071596/s1, Supplementary File S1: The mash distance tree and genomic similarity analysis supporting species classification.
