# Supplementary material for: Diagnostic Discordance During MDR-TB Treatment: Retrospective Identification of Mycobacterium avium Complex-Associated Nontuberculous Mycobacterium by Whole-Genome Sequencing
Source: Microorganisms. 2026 Jul 22;14(7):1596. doi: 10.3390/microorganisms14071596 (PMC13414386; doi:10.3390/microorganisms14071596)

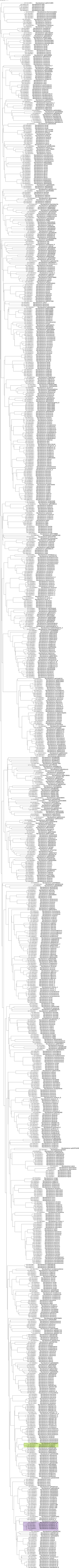

Supplement: Supplementary file 1 [file microorganisms-4264446-supplementary.pdf › microorganisms-4264446-supplementary.pdf]
